# Association between Sleep Quality and Periodontal Status: A Case-Control Study

**DOI:** 10.30476/dentjods.2024.101184.2278

**Published:** 2025-03-01

**Authors:** Fatemeh Kamalian Mehrizi, Ameneh Hosseini Yekani, Fahimeh Rashidi Maybodi

**Affiliations:** 1 Dental Student, School of Dentistry, Shahid Sadoughi University of Medical Sciences, Yazd, Iran; 2 Dept. of Oral Health and Community Dentistry, School of Dentistry, Shahid Sadoughi University of Medical Sciences, Yazd, Iran; 3 Dept. of Periodontics, School of Dentistry, Shahid Sadoughi University of Medical Sciences, Yazd, Iran

**Keywords:** Sleep Quality, Periodontal Diseases, Periodontitis, Case-control studies

## Abstract

**Statement of the Problem:**

In the literature, the relationship between sleep quality and periodontal diseases has been mentioned, but still there is a lack of consensus and a valid conclusion in the results obtained.

**Purpose:**

This study aimed to compare the sleep quality of patients with periodontitis and their healthy counterparts. The correlation between sleep quality score and age, gender, occupation, brushing pattern, and the severity of periodontal disease was also investigated.

**Materials and Method:**

This case-control study was conducted on 106 patients with periodontitis and 106 controls with healthy periodontium referring to the Periodontology Department of Yazd Dental School from December 2021 to April 2022. The sleep quality of the two groups was assessed by the Pittsburgh Sleep Quality Index (PSQI). Data were analyzed by ANOVA, t-test, and linear regression to assess possible correlations between the sleep quality score and demographic variables, tooth brushing pattern, and presence of periodontitis and its severity (alpha=0.05).

**Results:**

Totally, 149 females (70.3%) and 63 males (29.7%) with the mean age of 34.17±8.29 years, participated in this study. The sleep quality score had no significant correlation with age, gender, occupation, or tooth brushing pattern (*p*> 0.05). However, the sleep quality had a significant correlation with periodontitis (OR= 1.15, CI 95%: 1.02-1.29, *p*= 0.01). The sleep quality score had no significant correlation with the severity of periodontal disease (*p*= 0.225).

**Conclusion:**

Sleep quality of patients with periodontitis was significantly lower than that of healthy controls.

## Introduction

Periodontal disease is the most common cause of tooth loss worldwide, and has known adverse systemic effects [ 1 - 2 ]. Studies about the effect of risk factors on periodontal disease progression have focused on inflammatory reactions. These studies concluded that a regulated inflammatory host response is required for successful periodontal defense [ 1 , 3 ]. 

Sleep disorders and poor quality of sleep profoundly affect the physiological function of the human body, compromise immunity, enhance the inflammation process, and elevate the serum levels of inflammatory markers [ 4 - 5 ]. Insufficient sleep is an environmental stressor, which can make the host susceptible to microbial infections. It also increases the risk of periodontal disease through enhancement of inflammatory responses. Thus, periodontal disease may be correlated with sleep quality [ 6 ]. Insufficient sleep is a proven risk factor for progression of chronic diseases such as diabetes mellitus, cardiovascular diseases, and other inflammatory disorders. Evidence shows that proper sleep regulates the immune system and supports the immune function [ 7 - 8 ]. Acute and chronic sleep deprivation may trigger inflammatory processes, and increase the level of Creactive protein, peripheral blood circulation of leukocytes, and levels of interleukin (IL)-6, and tumor necrosis factor alpha [ 9 ]. Irwin *et al.* [ 10 ] showed higher levels of monocytes, IL-6, and tumor necrosis factor alpha in sleep-deprived individuals. Another study reported that even one night of sleep deprivation could induce the production of IL-1β and IL-1 receptor antagonist. It appears that insufficient sleep has a potential systemic inflammatory effect, which may susceptible the endothelial tissues to bacterial infection [ 11 ]. 

Since inflammation is a common characteristic of both periodontitis and insufficient sleep, insufficient sleep may play a role in increasing the risk of periodontal disease [ 12 - 14 ]. Singh *et al.* [ 15 ] reported that poor sleep quality was strongly correlated with chronic periodontitis. Karaaslan *et al.* [ 16 ] found a significant correlation between the severity of periodontitis and insufficient sleep and poor sleep quality. However, another study did not find any significant correlation between duration and quality of sleep with periodontal disease [ 17 ]. A systematic review by Muniz *et al.* [ 18 ] in 2021 also showed conflicting results for the association between sleep quality and periodontitis in literature and concluded that more studies are needed to confirm such association.

Considering the existing controversy regarding the correlation of sleep quality and periodontal disease, this study aimed to assess the sleep quality of periodontal patients and compare it with their healthy counterparts. Moreover, the possible association between sleep quality score and variables such as severity of periodontal disease, brushing pattern, and demographic factors were also investigated. 

## Materials and Method

This case-control study was conducted on 106 periodontitis patients and 106 controls with healthy periodontium referred to the Periodontology Department of Yazd Dental School between December 2021 until April 2022. The study protocol was approved by the Ethics Committee of the Shahid Sadougi University of Medical Sciences (IR.SSU.REC.1401.043). Informed consents were obtained from all participants prior to enrollment. This study conforms to the STROBE checklist (Table 1).

**Table 1:** STROBE Statement: checklist of items that should be included in reports of observational studies

	Item No.	Recommendation	Page No.
Title and abstract	1	(a) Indicate the study’s design with a commonly used term in the title or the abstract	1
		(b) Provide in the abstract an informative and balanced summary of what was done and what was found	1
Introduction			
Background/ rationale	2	Explain the scientific background and rationale for the investigation being reported	2
Objectives	3	State specific objectives, including any prespecified hypotheses	2
Methods			
Study design	4	Present key elements of study design early in the paper	1,2
Setting	5	Describe the setting, locations, and relevant dates, including periods of recruitment, exposure, follow-up, and data collection	3
Participants	6	(a) Cohort study—Give the eligibility criteria, and the sources and methods of selection of participants. Describe methods of follow-up	3
		Case-control study—Give the eligibility criteria, and the sources and methods of case ascertainment and control selection. Give the rationale for the choice of cases and controls	
		Cross-sectional study—Give the eligibility criteria, and the sources and methods of selection of participants	
		(b) Cohort study: For matched studies, give matching criteria and number of exposed and unexposed	3
		Case-control study—For matched studies, give matching criteria and the number of controls per case	
Variables	7	Clearly define all outcomes, exposures, predictors, potential confounders, and effect modifiers. Give diagnostic criteria, if applicable	3
Data sources/measurement	8*	For each variable of interest, give sources of data and details of methods of assessment (measurement). Describe comparability of assessment methods if there is more than one group	3
Bias	9	Describe any efforts to address potential sources of bias	3
Study size	10	Explain how the study size was arrived at	3
Continued on next page			
Quantitative variables	11	Explain how quantitative variables were handled in the analyses. If applicable, describe which groupings were chosen and why	3
Statistical methods	12	(a) Describe all statistical methods, including those used to control for confounding	4
		(b) Describe any methods used to examine subgroups and interactions	4
		(c) Explain how missing data were addressed	
		(d) Cohort study- If applicable, explain how loss to follow-up was addressed	3
		Case-control study- If applicable, explain how matching of cases and controls was addressed	
		Cross-sectional study- If applicable, describe analytical methods taking account of sampling strategy	
		(e) Describe any sensitivity analyses	----
Participants	13*	(a) Report numbers of individuals at each stage of study—e.g. numbers potentially eligible, examined for eligibility, confirmed eligible, included in the study, completing follow-up, and analyzed	4
		(b) Give reasons for non-participation at each stage	3
		(c) Consider use of a flow diagram	4
Descriptive data	14*	(a) Give characteristics of study participants (e.g. demographic, clinical, social) and information on exposures and potential confounders	4
		(b) Indicate number of participants with missing data for each variable of interest	3
		(c) Cohort study—Summarize follow-up time (e.g., average and total amount)	
Outcome data	15*	Cohort study—Report numbers of outcome events or summary measures over time	
		Case-control study—Report numbers in each exposure category, or summary measures of exposure	4,5
		Cross-sectional study—Report numbers of outcome events or summary measures	
Main results	16	(a) Give unadjusted estimates and, if applicable, confounder-adjusted estimates and their precision (e.g., 95% confidence interval). Make clear which confounders were adjusted for and why they were included	4,5
		(b) Report category boundaries when continuous variables were categorized	----
		(c) If relevant, consider translating estimates of relative risk into absolute risk for a meaningful time period	----
Continued on next page			
Other analyses	17	Report other analyses done—e.g. analyses of subgroups and interactions, and sensitivity analyses	----
Discussion			
Key results	18	Summarize key results with reference to study objectives	5
Limitations	19	Discuss limitations of the study, taking into account sources of potential bias or imprecision. Discuss both direction and magnitude of any potential bias	7
Interpretation	20	Give a cautious overall interpretation of results considering objectives, limitations, multiplicity of analyses, results from similar studies, and other relevant evidence	6,7
Generalizability	21	Discuss the generalizability (external validity) of the study results	6,7
Funding	22	Give the source of funding and the role of the funders for the present study and, if applicable, for the original study on which the present article is based	7,8

### Sample size

The sample size was calculated to be 212 participants assuming alpha=0.05, beta=0.1, S_1_=0.001, S_2_=0.28, X_1_=0.001, and X_2_=0.09 using the following equation: 

$n=\frac{\left(S_{1}^{2}+S_{2}^{2}\right){\left(Z_{1-\frac{\alpha }{2}}+Z_{1-\beta }\right)}^{2}}{{\left({\bar{X}}_{1}-{\bar{X}}_{2}\right)}^{2}}$

### Eligibility criteria

The inclusion criteria were age between 18 to 45 years, having at least 16 teeth, willingness for participation in the study, no smoking, no physical or mental disability, no pregnancy or nursing, no history of sleep apnea, no systemic disease, no history of periodontal therapy in the past 6 months, no use of antibiotics, anti-inflammatory drugs, sedatives or tranquilizers in the past 3 months, no intake of medications affecting the periodontal tissue, and absence of known causes of insomnia (such as unemployment, depression, bankruptcy, or bereavement). 

**Figure 1:**
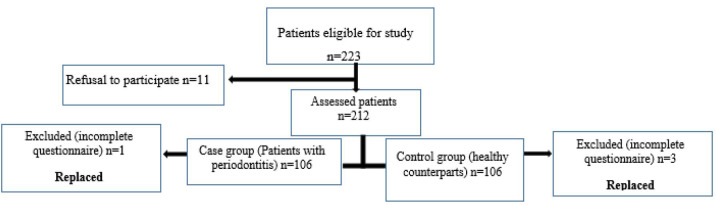
Flow diagram for patients' recruitment

The exclusion criterion was incomplete questionnaires. No data amputation was considered and incomplete questionnaires were replaced by new complete ones. Figure 1 shows the flow diagram for patients' recruitment. 

### Data collection

The study involved a clinical examination and a questionnaire. Variables such as demographic information, tooth-brushing pattern and the Pittsburgh Sleep Quality Index (PSQI) scores were included in the questionnaire and variables such as periodontal status and severity of periodontitis were assessed during full mouth examination.

During clinical examinations, patients were assessed using the 2017 world workshop classification of periodontal and peri-implant diseases [ 19 ]. Patients with gingival erythema, bleeding on probing, interdental clinical attachment loss in more than two non-adjacent teeth, or probing depth > 3mm in more than two teeth were diagnosed with periodontitis. Patients with stage I (mild periodontitis) to stage IV (advanced periodontitis), and grade B periodontitis (predictable progression in the absence of interfering factors such as systemic conditions and smoking) were selected and assigned to the case group [ 19 ]. Interdental clinical attachment loss, radiographic bone loss, and probing depth were all measured by one periodontist to eliminate the confounding effect of multiple examiners. 

A total of 106 age- and sex-matched controls with healthy periodontium were also selected and assigned to the control group. The flowchart of the selection of eligible patients into the study is shown in Figure 1. All participants in both groups filled out the PSQI, which is a valid, reliable and standard self-rated questionnaire [ 20 ], regarding the quality and pattern of sleep during a 1-month period. The Persian version of PSQI, which acceptable psychometric properties, was used (Cronbach's alpha coefficient was 0.77) [ 21 ]. Nineteen items of this questionnaire were grouped into seven domains (subjective sleep quality, sleep latency, sleep duration, habitual sleep efficiency, sleep disturbances, use of sleeping medications, and daytime dysfunction) and each scored on a 0 to 3 scale. By some means, use of sleep medications had already been controlled via inclusion criteria (not taking sedatives or tranquilizers in the past 3 months). All scores were then summed to produce a final score (range 0–21); higher PSQI scores indicate the worse quality of sleep [ 22 ]. 

### Statistical Analysis

Data were analyzed by ANOVA, t-test, and multiple linear regression to compare the mean score of sleep quality based on demographics, tooth brushing pattern (daily or irregular), type of occupation, having periodontitis or a healthy periodontium, and the severity of periodontal disease. All statistical analyses were carried out using SPSS version 22 (SPSS Inc., IL, USA) at 0.05 level of significance.

## Results

In total, 149 females (70.3%) and 63 males (29.7%) with a mean age of 34.17±8.29 years participated in this study. The mean age was 30.31±7.9 years in healthy control group and 38.04±6.7 years in the case group. Table 2 presents the demographic and general information of the participants. 

**Table 2:** Demographic and general information of the participants

Variable		Number	Percentage
Age	≤ 35 years	109	51.4
	>35 years	103	48.6
Gender	Female	149	70.3
	Male	63	29.7
Occupation	Housewife	121	57.1
	Government's employee	39	18.4
	Freelance (Non-Government)	52	24.5
Tooth brushing pattern	Yes	130	61.3
	No	82	38.7
Periodontal status	Healthy	106	50.0
	Mild periodontitis (stage I)	42	19.8
	Moderate periodontitis	35	16.5
	Advanced periodontitis (stage IV)	29	13.7

The mean sleep quality score was 4.5±2.52 in the control group and 5.18±3.22 in the case group. After controlling for the effect of age and gender by multiple linear regression model, the results showed significantly higher sleep quality score in the case group by 1.14 units, compared with the control group (*p*= 0.013). 

However, sleep quality score had no significant correlation with the severity of periodontal disease (*p*= 0.225) (Table 3). Age (*p*= 0.34) and gender (*p*= 0.92) had no significant correlation with the sleep quality score either (Table 4). Daily tooth brushing (*p*= 0.91), and occupation (*p*= 0.27) were not significantly correlated with the sleep quality score (Table 4). 

**Table 3:** Mean sleep quality score based on the severity of periodontal disease

Periodontal status	Sleep quality score		*p* Value*
	Mean	Std. deviation	
Healthy	4.5	2.52	0.225*
Mild periodontitis (stage I)	5.11	2.34	
Moderate periodontitis	6.1	3.79	
Advanced periodontitis (stage IV)	4.1	3.33	

* ANOVA

**Table 4:** Mean sleep quality score based on age, gender, tooth brushing pattern and occupation

Variable	Category	Sleep quality score		*p* Value
		Mean	Std. deviation	
Age	≤35 yrs.	5.02	2.75	0.34*
	>35 yrs.	4.65	3.06	
Gender	Female	4.83	2.69	0.92*
	Male	4.87	3.39	
Tooth brushing pattern	Daily	4.86	2.79	0.91*
	Irregular	4.81	3.1	
Occupation	Housewife	5.06	2.84	0.27**
	Freelance	4.8	3.39	
	Government's employee	2.34	4.2	

* T-test;

** ANOVA

## Discussion

This study assessed the sleep quality of periodontal patients. After controlling for the effect of age and gender, a significant correlation was found between the PSQI score and periodontitis, such that periodontal patients acquired a higher score, indicating lower sleep quality. This finding was in agreement with the results of Singh *et al.* [ 15 ], Alhassani and Al-Zahrani [ 23 ], Romandini *et al.* [ 24 ], Grover *et al.* [ 3 ], Karaaslan and Dikilitaş [ 16 ], and Wiener [ 25 ]. However, precise comparison of the results is difficult since some studies such as studies by Alhassani and Al-Zahrani [ 23 ], and Wiener [ 25 ] considered the duration of sleep (> or < 7 hours) as the criterion for sleep quality instead of using the PSQI. Alqaderi *et al.* [ 26 ] pointed to the correlation of poor quality of sleep and periodontitis; however, they assessed the sleep duration (< or > 7 hours) rather than quality. In addition, they only evaluated patients with severe periodontitis and did not exclude diabetic patients. They reported stronger correlation of periodontitis with poor sleep quality in diabetic patients, which may be due to the correlation of insomnia with glucose intolerance and insulin resistance [ 26 ]. Islam *et al.* [ 17 ] used the Japanese version of the PSQI and found no significant correlation between the sleep quality score and periodontitis, which was different from the present findings. 

The present study found no significant correlation between the severity (stage) of periodontitis and sleep quality. However, it should be noted that only grade B patients were enrolled since those with systemic diseases and cigarette smoking were excluded. The results of Karaaslan *et al.* [ 16 ] were different from the present findings, despite the selection of stage and grading of periodontitis; such that the poorest sleep quality (highest score), was significantly correlated with the most severe stage (IV) and grade (C) of periodontitis. Tawfig *et al.* [ 27 ] pointed to the significant correlation of higher PS-QI scores with higher stages of periodontitis but not with grade of periodontitis. It should be noted that their control group included periodontally healthy and gingivitis patients that was different from the control group of the present study. Grover *et al.* [ 3 ] demonstrated an ascending trend for the PSQI score of controls with healthy periodontium, gingivitis patients, and periodontitis patients; they interpreted it as a correlation between the severity of periodontal disease and sleep quality. Difference between their results and the present findings may be due to different grouping of participants and interpretation of the results. Al-Zahrani *et al.* [ 28 ] pointed to the correlation of poorer oral health and lower sleep quality; however, they used the lower number of residual teeth as the criterion for lower oral health. Since lower mastication efficiency has been reported to be correlated with lower sleep quality [ 29 ], having at least 16 teeth was an inclusion criterion for the present study. 

In the current study, age had no significant correlation with the sleep quality score. This correlation has not been addressed in any previous study on the relationship of periodontitis and sleep quality [ 17 , 23 , 26 ]. Thus, further studies are warranted on this correlation. 

The present results showed no significant correlation between gender and sleep quality, which was in contrast to the results of Karagozoglu and Bingöl [ 30 ] who reported higher sleep quality in males, but was in agreement with the results of Atadokht [ 31 ]. No previous study was found on the correlation of periodontitis and sleep quality to address the effect of gender as well, which calls for further studies in this regard. 

Daily tooth brushing, irrespective of periodontal status and occupation of patients, had no significant correlation with their sleep quality in the present study. No previous study was found in this regard to compare our results with. Adherence to daily brushing may not necessarily be interpreted as good oral hygiene status. Because the method of brushing and the time spent are also important in the amount of plaque removal.

It should be noted that a number of confounders might affect the sleep quality such as nutrition, stress, undetected psychological conditions, and mood, among others, which were not taken into account in the present study. Thus, interpretation and generalization of the results should be done with caution. In addition, this study had a cross-sectional design, and only showed simultaneous presence of periodontitis and poor sleep quality in the case group, suggesting that treatment of periodontitis may improve the sleep quality. However, a causal relationship could not be established in the present study due to its cross-sectional design, and longitudinal studies are required to better elucidate this relationship. 

Future studies should perform interventions to improve sleep quality in periodontitis patients, and assess their possible effect on periodontal parameters after standardization of demographic factors such as age, gender, and socioeconomic status. 

## Conclusion

The results showed that, after adjusting the analysis for age and gender, the sleep quality of periodontal patients was significantly lower than that of healthy individuals. Further research to confirm the role of sleep, including the use of subjective and objective measures to determine sleep pattern, is necessary to better understand the mechanism of the potential association between sleep quality and periodontitis.

### Limitations

A case-control design and a comprehensive clinical examination of the whole mouth were included to strengthen the obtained results. However, some unknown confounding factors may affect sleep quality and periodontitis.
